# GeS_2_–In_2_S_3_–CsI Chalcogenide Glasses Doped with Rare Earth Ions for Near- and Mid-IR Luminescence

**DOI:** 10.1038/srep37577

**Published:** 2016-11-21

**Authors:** Legang Li, Junyi Bian, Qing Jiao, Zijun Liu, Shixun Dai, Changgui Lin

**Affiliations:** 1 Laboratory of Infrared Materials and Devices, The Research Institute of Advanced Technologies, Ningbo University, Ningbo 315211, China; 2 Key Laboratory of Photoelectric Detection Materials and Devices of Zhejiang Province, Ningbo University, Ningbo 315211, China

## Abstract

Chalcogenide glass has been considered as a promising host for the potential laser gain and amplifier media operating in near- and mid-IR spectral region. In this work, the IR luminescence spectra of rare earth ions (Tm^3+^, Er^3+^, and Dy^3+^) doped 65GeS_2_–25In_2_S_3_–10CsI chalcogenide glasses were measured under the excitation of an 808 nm laser diode. To the best of our knowledge, it firstly provides the luminescence spectra of a full near- and mid-IR spectral range from 1 to 4 μm in rare earth ions doped chalcogenide glasses. The results of absorption spectra, luminescence spectra, and fluorescence decay curves were obtained in these samples with singly-, co- and triply-doping behaviors of Tm^3+^, Er^3+^, and Dy^3+^ ions. In order to search possible efficient IR emissions, the luminescence behavior was investigated specifically with the variation of doping behaviors and dopant ions, especially in the samples co- and triply-doped active ions. The results suggest that favorable near- and mid-IR luminescence of rare earth ions can be further modified in chalcogenide glasses through an elaborated design of doping behavior and optically active ions.

In order to obtain IR luminescence of new wavelengths or bandwidths, glasses doped with different rare earth ions (REIs), especially Dy^3+^, Er^3+^, and Tm^3+^, have been intensively investigated in the past decades. For instance, the 1.3 μm emission of Dy^3+^ ion has been well adopted for the applications of optical communication^1^. The broadband optical amplification beyond the conventional 1.54 μm window of Er^3+^-doped fiber amplifier is of great interests for the increasing demands of information traffic^2^. To further extend the bandwidth of the light sources, one of the effective approaches is to co-dope with other active ions, such as Tm^3+^, through the overlap of emission bands. The ^3^H_4_ → ^3^H_6_ transition of Tm^3+^ ions generates the emission around 1460 nm, which could provide an excellent complement for the ^4^I_11/2_ → ^4^I_13/2_ transition (1540 nm) of Er^3+^ and the ^6^F_11/2_ + ^6^H_9/2_ → ^6^H_15/2_ transition (1328 nm) of Dy^3+^ ions. Thus the Tm^3+^-Dy^3+^ co-doped chalcohalide glasses have been recognized as one of the promising candidate materials for fiber-amplifiers and IR laser devices^3^. Meanwhile, it has been shown that an Er^3+^-Tm^3+^ co-doped 20 m long silica fiber amplifier generates spontaneous emission with bandwidth over 90 nm (1460–1550 nm), if it is pumped at 980 nm^4^. Nevertheless, the radiative transition from ^3^H_4_ to ^3^H_5_ (∼4300 cm^−1^) of Tm^3+^ is absent in silica or silicate glasses due to their relatively high maximum phonon energy (MPE, ∼1100 cm^−1^)^4^. Thus in order to obtain the laser gain and amplifier media operating in near- and mid-IR spectral region, much efforts have been devoted into searching low MPE materials, such as chalcogenide glasses (<350 cm^−1^)^5^, fluoride glasses (∼550 cm^−1^)^6^, tellurite glasses (∼750 cm^−1^)^7^,^8^, and oxyfluoride glass-ceramics^9^. Among them, chalcogenide glass is distinguished due to its nature of the lowest MPE.

Since the rediscovery of the excellent mid-IR transmission property of As_2_S_3_ in 1950s^10^, chalcogenide glasses are well known because of their unique properties of broad IR transparency window, semiconductivity, photosensitivity, and fast ionic conduction. The IR transmission region extending from 12 to 20 μm, depending on the chalcogen elements of S, Se, and Te, makes them favorable in the applications of IR optics, e.g. the lens for thermal imaging. Besides, compared with that of oxide and fluoride glasses, they also have the lowest MPE, which plays an important role in bridging the small energy gaps for the radiative transitions^11^. It is of great significance to explore new photonic properties of active ions, especially for the IR luminescence^12^,^13^.

Among the numerous investigation of REIs doped chalcogenide glasses, GeS_2_–Ga_2_S_3_ glasses have been well studied because of the augmented REIs solubility, which is originated from the structural modification caused by gallium^14^. By considering the similarity of chemical properties between indium and gallium, GeS_2_–In_2_S_3_ glasses also have been evidenced to possess the similar property of high REI solubility akin to that of gallium glasses^15^,^16^. Here, GeS_2_–In_2_S_3_ based glasses were specially selected because of the other superior optical properties, e.g. high refractive index. Meanwhile, CsI was introduced for the following two reasons. First, good glass-forming ability could be achieved due to the large radius of cesium ion, which can stabilize the formed complex anion in the glassy network^17^. Second, the addition of iodine ions results in a broadening of optical bandgap and the consequent shift of the visible cut-off edge towards shorter wavelengths^18^, which would benefit for the selection of pump sources during luminescence measurements. Thus, in this report, chalcogenide glasses with the composition of GeS_2_–In_2_S_3_–CsI are selected as the hosts for doping three different REIs of Dy^3+^, Er^3+^, and Tm^3+^. The IR luminescence spectra ranging from 1 to 4 μm will be measured and the optical transitions of the singly-, co-, or triply-doping of REIs will also be discussed in detail. To the best of our knowledge, it might be the first work to present such full spectral region of near- and mid-IR luminescence in glassy materials.

## Results and Discussion

**Figure 1:**
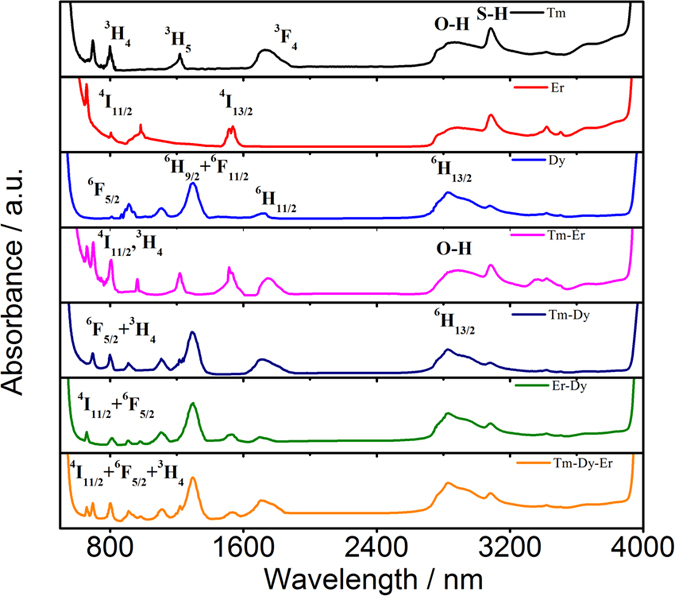
The absorption spectra of singly-, co-, and triply-doped 65GeS_2_–25In_2_S_3_–10CsI glass (thickness of 2 mm) at room temperature.

Figure 1 shows the absorption spectra of REIs singly-, co- and triply-doped 65GeS_2_–25In_2_S_3_–10CsI glass at room temperature in the wavelength region of 400–4000 nm. Some of the absorption bands in IR region are attributed to the impurities of O-H (~2800 nm), S-H (~3100 nm), and others (~3400 nm and ~3500 nm), respectively. Most of the excited states are labeled in Fig. 1, expect some of them with weak absorbance, e.g. ^4^I_9/2_ of Er^3+^ centered at 804 nm. For the Tm^3+^-doped glass sample, a strong absorption band of the excited state ^3^H_4_ locates around 798 nm. In the Er^3+^-Tm^3+^ co-doped sample, the absorption band centered at 806 nm is enhanced due to the very closer transitions of Er^3+^: ^4^I_15/2_ → ^4^I_9/2_ and Tm^3+^: ^3^H_6_ → ^3^H_4_. It indicates that the higher pumping efficiency could be realized compare to that of the Er^3+^ singly-doped glass sample. The absorption bands corresponding to internal 4f–4 f electronic transitions of Dy^3+^-doped are also indicated in the Fig. 1. The absorption peaks of around 914, 1114, 1298, 1724, and 2833 nm are obvious, whereas that of 808 nm is very weak, suggesting the pumping efficiency under the excitation of 808 nm LD will be very low in this sample. It is seen that all of the co- or triply-doped Ge-In-S-CsI glass show the combined absorption bands of the respective REIs in the spectra. The fluorescence spectra of the singly-, co-, or triply-doped samples will be displayed and analyzed in detail in the following.

### Singly- and co-doping of Er^3+^ and Tm^3+^

**Figure 2:**
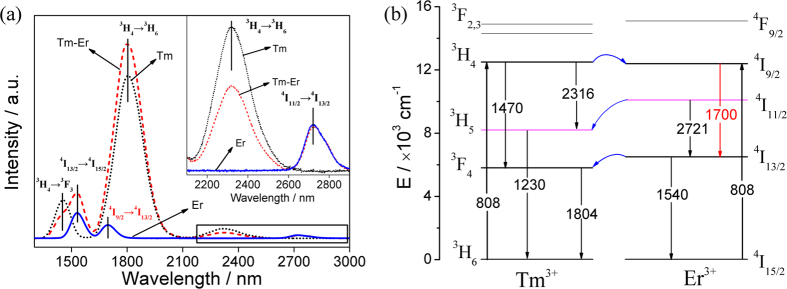
(**a**) The IR fluorescence spectra under the excitation of 808 nm and (**b**) energy transfer scheme of 65GeS_2_–25In_2_S_3_–10CsI glasses with the various doping behavior of Er^3+^-, Tm^3+^-, and Tm^3+^-Er^3+^.

Figure 2a shows the IR fluorescence spectra of the 65GeS_2_–25In_2_S_3_–10CsI glasses doped with Tm^3+^ and Er^3+^ in different doping behavior. There have 5 emission bands located at 1470, 1540, 1804, 2316 and 2721 nm, respectively. As indicated in Fig. 2b, the transitions of Tm^3+^: ^3^H_4_ → ^3^F_4_, Tm^3+^: ^3^H_4_ → ^3^H_5_ and Er^3+^: ^4^I_11/2_ → ^4^I_13/2_ contribute to the emissions located at 1470, 2316, and 2721 nm. The excited levels of Tm^3+^:^3^F_4_ and Er^3+^: ^4^I_13/2_ relaxes to the respective ground states of ^3^H_6_ and ^4^I_15/2_ generating the 1804 nm and 1540 nm emissions, respectively. Besides, in the Er^3+^ singly-doped samples, there has a band centered at 1700 nm which is originated from the transition of Er^3+^: ^4^I_9/2_ → ^4^I_13/2_. It is obvious that the 1540 nm emission of Er^3+^ singly-doped samples attributed to ^4^I_13/2_ → ^4^I_15/2_ transition has a full width at half-maximum (FWHM) of ∼80 nm. It can be further improved through the co-doping of Er^3+^-Tm^3+^, which shows an emission ranging from 1350 to 1600 nm with a FWHM of ∼140 nm, which is much broader than that of Er^3+^-Tm^3+^ co-doped silica fiber (90 nm)^19^.

As shown in Fig. 2b, the energy transfer between Tm^3+^ and Er^3+^ can be achieved through several shortcuts, which would lead to the variation of emission behavior. As shown in Fig. 2a, the intensity of 1804 nm emission is enhanced by co-doping with Er^3+^, whereas that of the 1470 nm and 2316 nm emissions are decreased. These can be ascribed to the small energy gap between Tm^3+^: ^3^H_4_ and Er^3+^: ^4^I_9/2_, which contributes to a resonant energy transfer for Er^3+^: ^4^I_9/2_ level by depopulating the Tm^3+^: ^3^H_4_ level. Although there exists energy transfer from Er^3+^: ^4^I_13/2_ to Tm^3+^: ^3^F_4_, the intensity of 1540 nm emission increases by 79% in Er^3+^-Tm^3+^ co-doped sample. It suggests that this co-doped sample has a better pumping efficiency and the sensitized effect of Tm^3+^ ions is stronger than the energy transfer process.

All emission lifetimes and the related parameters of the above-mentioned samples are calculated and collected in Table 1. The decay profiles were recorded by NIR-PMT (Hamamatsu R5509-72 Photomultiplier) and InSb detectors by monitoring of the emissions at 1–2 μm and 2–4 μm, respectively. The lifetime values are also related to the energy transfer processes, akin to the emission intensity. In Tm^3+^ singly and Tm^3+^-Er^3+^ co-doped samples, the emission lifetimes decrease from 172 to 156 μs for 1470 nm (^3^H_4_ → ^3^F_4_) and from 177 to 175 μs for 2316 nm (^3^H_4_ → ^3^H_5_), while it increases from 1189 to 1245 μs for 1804 nm (^3^F_4_ → ^3^H_6_). It is worthy to note that the emission at 1540 nm of the Er^3+^-doped sample become stronger by the co-doping with Tm^3+^ ions, indicating a higher pumping efficiency. The energy transfer efficiency of the studied samples are collected in Supplementary Table S1.

**Figure 3:**
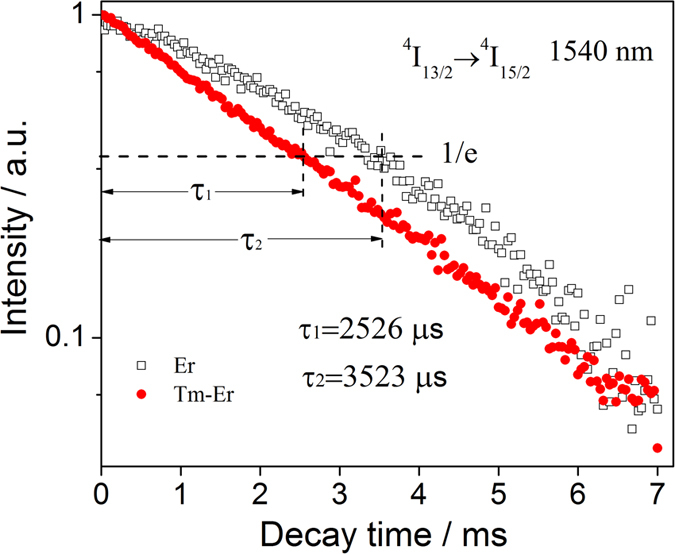
Decay curves of ^4^I_13/2_ → ^4^I_15/2_ emission (1540 nm) in Er^3+^ doped and Tm^3+^-Er^3+^ co-doped 65GeS_2_–25In_2_S_3_–10CsI glasses.

Figure 3 shows the typical fluorescence decay curves of Er^3+^-doped and Tm^3+^-Er^3+^ co-doped glasses, which were recorded by monitoring the transition of ^4^I_13/2_ → ^4^I_15/2_ (1540 nm) as displayed in Fig. 2b. The lifetime here is obtained according to the definition of the time where the emission intensity decreases to 1/*e* of its initial value. The lifetime of 1540 nm emission in co-doped sample decreases from 3523 to 2526 μs. The process is satisfied by the energy transfer from Er^3+^: ^4^I_13/2_ to Tm^3+^: ^3^F_4_. There is a particular shortcut that energy transfer from Er^3+^: ^4^I_11/2_ to Tm^3+^: ^3^H_5_. The lifetime of 2721 nm emission does not change significantly from 2159 to 2069 μs (within the error limit of ±5%). The variation of emission intensity and lifetime of 2721 nm are nearly consistent. Nevertheless, the effect of Tm^3+^ doping behavior on the 2721 nm emission of Er^3+^ should be further investigated and checked by the concentration variation.

The energy transfer efficiency from Er^3+^- to Tm^3+^-Er^3+^ co-doped can be calculated by the following equation^20^


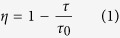


where 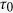 and 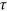 are the lifetimes of ^4^I_13/2_ → ^4^I_15/2_ emission (1540 nm) in Er^3+^-doped and Tm^3+^-Er^3+^co-doped samples (the lifetimes in the absence and presence of acceptors), respectively. The lifetime values of ^4^I_13/2_ → ^4^I_15/2_ in these singly- and co-doped samples are marked in Fig. 3. The lifetime reduces strongly by co-doping with Tm^3+^, indicating the efficient energy transfer from Er^3+^ to Tm^3+^. In the co-doped sample, the energy transfer efficiency from Er^3+^ to Tm^3+^ is ~28.2%, which is different from that of ~80% reported in TeO_2_–WO_3_–PbO glass^20^ and tellurite fiber^7^,^21^. In this case, the low energy transfer efficiency is favorable to the interested broadband emission around 1540 nm.

In this doping behavior, for the near-IR emission of 1540 nm (Er^3+^: ^4^I_13/2_ → ^4^I_15/2_), the co-doping of Tm^3+^ can effectively extend its FWHM and intensity in the 65GeS_2_–25In_2_S_3_–10CsI chalcogenide glass. It is due to the significant energy transfer process between Tm^3+^ and Er^3+^. Thus, this Tm^3+^-Er^3+^co-doped sample has a broad bandwidth with a flat-gain spectrum for minimizing the channel-to-channel gain excursion and crosstalk in a high-speed network.

### Singly- and co-doping of Dy^3+^ and Tm^3+^

**Figure 4:**
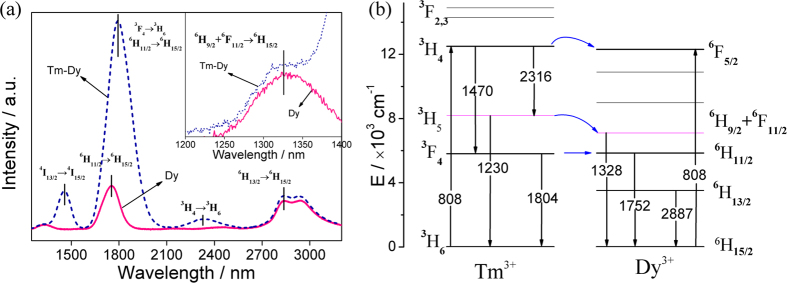
(**a**) The IR fluorescence spectra and (**b**) energy transfer scheme of 65GeS_2_–25In_2_S_3_–10CsI glasses doped with Dy^3+^- and Tm^3+^-Dy^3+^, respectively.

Figure 4 shows the IR fluorescence spectra of Dy^3+^-doped and Tm^3+^-Dy^3+^ co-doped glasses in the ranges from 1200 to 3300 nm and also the corresponding energy-level diagram. In Dy^3+^-doped glass, three major emission bands are observed around 1328, 1752 and 2887 nm. As shown in Fig. 4b, they are ascribed to the transitions of ^6^F_11/2_ + ^6^H_9/2_ → ^6^H_15/2_, ^6^H_11/2_ → ^6^H_15/2_, and ^6^H_13/2_ → ^6^H_15/2_, respectively. As indicated in Fig. 4b, three shortcuts of the energy transfer are existed between Tm^3+^ and Dy^3+^. The first shortcut of the energy transfer from Tm^3+^: ^3^H_4_ to Dy^3+^: ^6^F_5/2_ leads to that the emission intensities and lifetimes of 1470 and 2316 nm decrease as displayed in Table 1. The energy transfer from Tm^3+^: ^3^H_5_ to Dy^3+^: ^6^F_11/2_ + ^6^H_9/2_ results in an overlapping emission from Dy^3+^ (1328 nm) to Tm^3+^ (1470 nm), a 17% increased emission at the 1328 nm (as shown in the inset of Fig. 4a), and a elongated lifetime from 41 to 68 μs. In addition, the two energy levels of Dy^3+^ (1752 nm, ^6^H_11/2_ → ^6^H_15/2_) and Tm^3+^ (1804 nm, ^3^F_4_ → ^3^H_6_) are very close, resulting in the energy transfer from Tm^3+^: ^3^F_4_ to Dy^3+^: ^6^H_11/2_. Thus the emission peak of the co-doped sample is shifted to longer wavelength (1794 nm), and the emission intensity enhances. Furthermore, the energy transfer from Tm^3+^: ^3^F_4_ to Dy^3+^: ^6^H_11/2_ can populate the initial state of the 2887 nm emission^22^, then contribute to the 18% increase of the 2887 nm emission intensity in Tm^3+^-Dy^3+^ co-doped sample.

**Figure 5:**
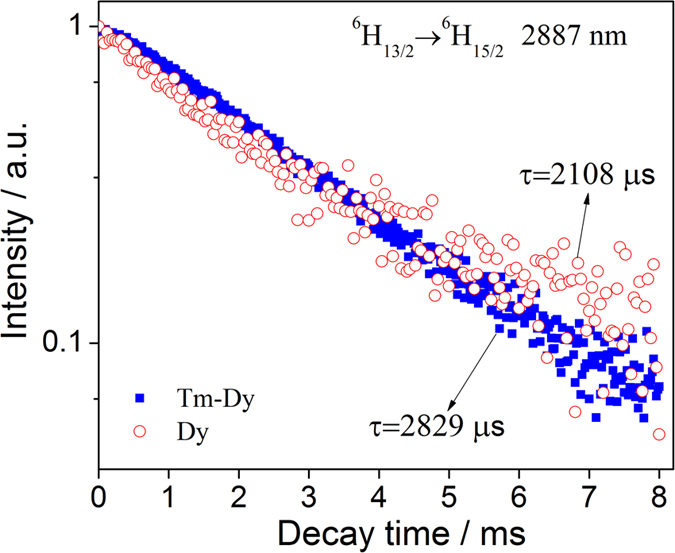
Decay curves of ^6^H_13/2_ → ^6^H_15/2_ transition (2887 nm) in Dy^3+^-doped and Tm^3+^-Dy^3+^co-doped 65GeS_2_–25In_2_S_3_–10CsI glasses.

The decay profiles of ^6^H_13/2_ → ^6^H_15/2_ transition in Dy^3+^-doped and Tm^3+^-Dy^3+^ co-doped samples are shown in Fig. 5. And the lifetime values are collected in Table 1. It is obvious that the intensities of 2887 nm emission vary slightly in singly- and co-doped samples, whereas their lifetimes change intensively from 2108 to 2829 μs.

In this doping behavior, the Dy^3+^ singly- and Dy^3+^-Tm^3+^ co-doped samples have three main peaks in near- and mid-IR spectral region. In particular, the 2887 nm emission always exists, which is better than that observed in Ge-Ga-S-CdI_2_ chalcohalide glasses^3^, indicating that the host glass is a good choice for doping Dy^3+^ ions. Furthermore, as indicated by the emission intensity and lifetime, we can see that the co-doping of Tm^3+^ ions is favorable to the emission of Dy^3+^ ions in the 2–4 μm region.

### Singly- and co-doping of Er^3+^ and Dy^3+^

**Figure 6:**
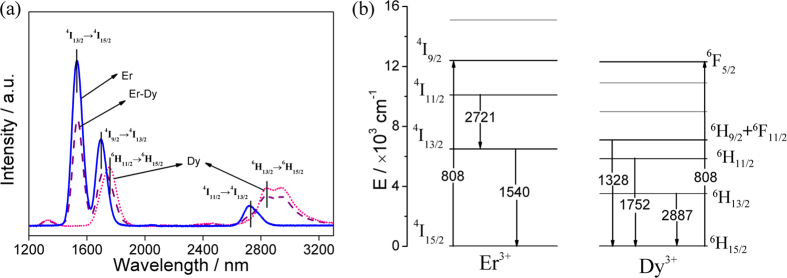
(**a**) The IR fluorescence spectra and (**b**) energy transfer scheme of 65GeS_2_–25In_2_S_3_–10CsI glasses doped with Er^3+^-, Dy^3+^-, and Er^3+^-Dy^3+^, respectively.

Figure 6a shows the fluorescence spectra of the (Er^3+^ and Dy^3+^) singly- and co-doped samples and the corresponding energy-level diagram. It is obvious that four main emission bands are located at 1540, 1752, 2721 and 2887 nm in the Dy^3+^ doped sample, and they decline with the co-doping of Er^3+^. It is in good accordance with the variation of the absorption bands in Fig. 1.

**Figure 7:**
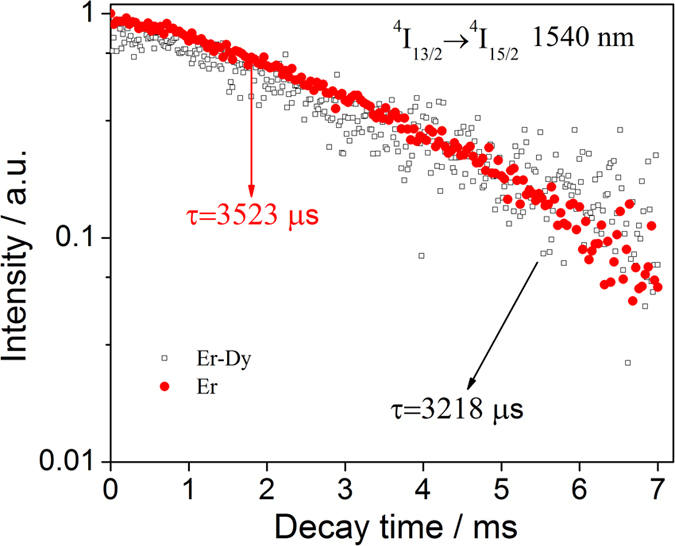
Decay curves of ^4^I_13/2_ → ^4^I_15/2_ emission (1540 nm) in Er^3+^-doped and Er^3+^-Dy^3+^co-doped 65GeS_2_–25In_2_S_3_–10CsI glasses.

From the decay curve in Fig. 7, the lifetime of the 1540 nm emission in Er^3+^-Dy^3+^ co-doped sample is equal to 3218 μs (listed in Table 1). In comparison to the above results, it suggests that the energy transfer efficiency of Er^3+^-Dy^3+^ co-doped sample is far less than that of Tm^3+^-Er^3+^ and Tm^3+^-Dy^3+^ co-doped ones. The co-doping of these two REIs (Dy^3+^ and Er^3+^) are not suitable for the near- and mid-IR emissions in chalcogenide glasses, because the co-doped sample shows a weakening effect on each peak.

### Singly-, co- and triply-doping of Er^3+^, Dy^3+^, and Tm^3+^

**Figure 8:**
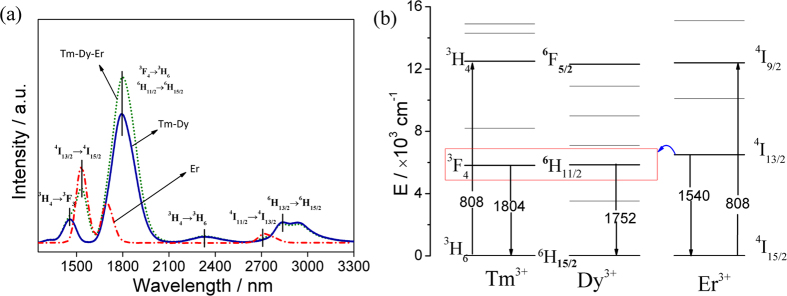
(**a**) The IR fluorescence spectra and (**b**) energy transfer scheme of 65GeS_2_–25In_2_S_3_–10CsI glasses with various doping behavior of Er^3+^, Tm^3+^-Dy^3+^, and Tm^3+^-Dy^3+^-Er^3+^.

The fluorescence spectrum of Tm^3+^-Dy^3+^-Er^3+^ triply-doped 65GeS_2_–25In_2_S_3_–10CsI glass is shown in Fig. 8a. A broadband emission from 1300 nm to 1600 nm is observed, which includes the transitions of Dy^3+^ (1328 nm, ^6^F_11/2_ + ^6^H_9/2_ → ^6^H_15/2_), Tm^3+^ (1470 nm, ^3^H_4_ → ^3^F_4_) and Er^3+^ (1540 nm, ^4^I_13/2_ → ^4^I_15/2_). The emission intensity of the triply-doped sample is improved in comparison with that of Er^3+^-doped and Tm^3+^-Dy^3+^ co-doped samples.

**Figure 9:**
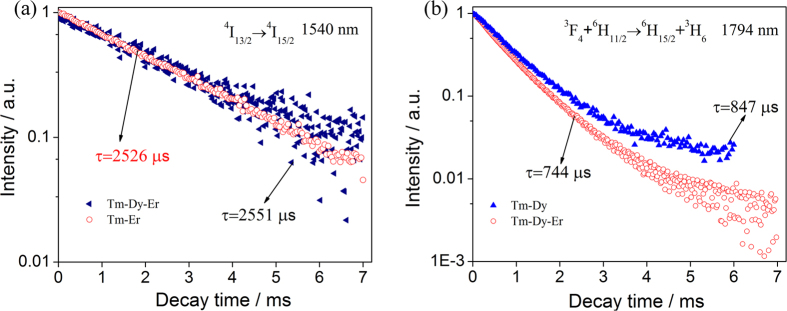
Decay curves of (**a**) the 1540 nm emission (Er^3+^: ^4^I_13/2_ → ^4^I_15/2_) in Tm^3+^-Er^3+^ co-doped and Tm^3+^-Dy^3+^-Er^3+^ triply-doped samples, and (**b**) the 1794 nm emission (Dy^3+^: ^6^H_11/2_ → ^6^H_15/2_ and Tm^3+^: ^3^F_4_ → ^3^H_6_) in Tm^3+^-Dy^3+^ co-doped and Tm^3+^-Dy^3+^-Er^3+^ triply-doped 65GeS_2_–25In_2_S_3_–10CsI glasses.

Figure 9 shows the decay profiles of the 1540 nm emission (Er^3+^: ^4^I_13/2_ → ^4^I_15/2_) in Tm^3+^-Er^3+^ co-doped and Tm^3+^-Dy^3+^-Er^3+^ triply-doped samples, and the 1794 nm emission (Dy^3+^: ^6^H_11/2_ → ^6^H_15/2_ and Tm^3+^: ^3^F_4_ → ^3^H_6_) in Tm^3+^-Dy^3+^ co-doped and Tm^3+^-Dy^3+^-Er^3+^ triply-doped 65GeS_2_–25In_2_S_3_–10CsI glasses. The lifetime of Er^3+^: ^4^I_13/2_ → ^4^I_15/2_ transition (1540 nm) in Tm^3+^-Er^3+^-Dy^3+^ triply-doped sample is 2551 μs (listed in Table 1), which is close to that of Tm^3+^-Er^3+^ co-doped one as displayed in Fig. 9a. According to the lifetime values marked in Fig. 9a, the energy transfer efficiency of the triply-doped sample is as high as 27.5%, it is compared to ~28.2% in Tm^3+^-Er^3+^ co-doped one. For the 1794 nm emission, there has a special shortcut of the energy transfer from Er^3+^: ^4^I_13/2_ to Tm^3+^: ^3^F_4_ and Dy^3+^: ^6^H_11/2_ as indicated in Fig. 8b. Finally, the reduction and improvement of the studied emissions are concluded in Supplementary Table S2.

In this work, the triple doping in 65GeS_2_–25In_2_S_3_–10CsI glass is of a certain research value, not only because it remains a appropriate energy transfer efficiency, but also it is beneficial to the IR emissions at desirable wavelengths through the energy transfer process. To the best of our knowledge, this work is the first one to present such broad IR emission behavior in REIs doped chalcogenide glasses.

## Conclusions

Near- and mid-IR luminescence spectra of Er^3+^, Tm^3+^, and Dy^3+^ ions was recorded in 65GeS_2_–25In_2_S_3_–10CsI glasses ranging from 1 to 4 μm with different doping behavior, e.g. singly-, co-, and triply-doping. The experimental results show that the co-doping of Tm^3+^ could greatly improve the pump efficiency of Dy^3+^ and Er^3+^ ions, and their fluorescence intensities. The Tm^3+^-Er^3+^ and Tm^3+^-Dy^3+^ co-doped samples present superior spectroscopic properties that is beneficial for the interested wavelength within 1–4 μm region. In addition, it provides the first luminescence investigation in Er^3+^-Tm^3+^-Dy^3+^ triply doped chalcogenide glasses. The energy transfer efficiency of the triple-doped sample is as high as Tm^3+^-Er^3+^ co-doped one, due to the energy transfer from Er^3+^ ions to Tm^3+^-Dy^3+^ ion. Although the efficient mid-IR laser is still absent in chalcogenide glasses, this work provide a full set of IR luminescence data available for the REIs doped chalcogenide glasses. It would be of significant guidance for the future development in this research field.

## Method

Glass samples of 65GeS_2_–25In_2_S_3_–10CsI singly-, co-, and triply-doped with 0.1 mol% REIs (Tm, Dy_2_S_3_, and Er) were synthesized by melting the mixtures of constituent elements (Ge, In, S, and CsI of 99.999%) in evacuated (~10^−3^ Pa) and flame-sealed silica ampoule. The batches were melted at 990 °C for 22 h, and then quenched in a saturated brine. The obtained glassy samples were annealed at 310 °C for 6 h. Glass rods were obtained by taking them out from the ampoules and finally cut into discs (Ø 9 mm × 2 mm), which were then polished for succeeding experiments.

The absorption spectra were recorded by a UV/VIS/NIR spectrophotometer (PERKIN-ELMER-LAMBDA 950) and FTIR spectroscopy (Thermo Scientific Nicolet 380) in the spectral ranges of 400–2500 nm and 2500–4000 nm, respectively. Excitation and emission spectra were measured by employing a fluorescence spectrometer (Edinburgh, ENGLAND FLS980). Fluorescence spectra were obtained using 808 nm excitation from a laser diode (LD) (the average power is 1 W). NIR-PMT and InSb detectors cooled with liquid nitrogen were employed to record the near- and mid-IR fluorescence from 1100 to 3300 nm. The fluorescence decay curves were recorded with InSb detector by monitoring the interested IR emissions. To make the results comparable, a fixed configuration between the sample, laser, and detector is employed and carefully used. By checking the variation of emission data in several measurements for the same sample, the error of these results are within ±5%. All the measurements were performed at room temperature.

## Additional Information

**How to cite this article**: Li, L. *et al*. GeS_2_–In_2_S_3_–CsI Chalcogenide Glasses Doped with Rare-Earth Ions for Near- and Mid-IR Luminescence. *Sci. Rep*. **6**, 37577; doi: 10.1038/srep37577 (2016).

**Publisher’s note**: Springer Nature remains neutral with regard to jurisdictional claims in published maps and institutional affiliations.

## Supplementary Material

Supplementary Information

## Figures and Tables

**Table 1:** Fluorescence decay curves of all samples and their energy transfer efficiency.

Sample	Transition	Wavelength/nm	τ/μs	Correlation coefficient of fit/%	Transfer efficiency/%
Tm^3+^-doped	^3^H_4_ → ^3^F_4_	1470	172	99.8	
	^3^F_4_ → ^3^H_6_	1804	1189	99.9	
	^3^H_4_ → ^3^H_5_	2316	177	99.1	
Er^3+^-doped	^4^I_13/2_ → ^4^I_15/2_	1540	3523	99.1	
	^4^I_11/2_ → ^4^I_13/2_	2721	2159	99.1	
Tm^3+^-Er^3+^	^3^H_4_ → ^3^F_4_	1470	156	99.9	
	^4^I_13/2_ → ^4^I_15/2_	1540	2526	99.9	28.2
	^3^F_4_ → ^3^H_6_	1804	1245	99.9	
	^3^H_4_ → ^3^H_5_	2316	175	99.2	
	^4^I_11/2_ → ^4^I_13/2_	2721	2065	98.3	
Dy^3+^-doped	^6^H_9/2_ + ^6^F_11/2_ → ^6^H_15/2_	1328	41	96.7	
	^6^H_11/2_ → ^6^H_15/2_	1752	501	99.6	
	^6^H_13/2_ → ^6^H_15/2_	2887	2108	97.4	
Tm^3+^-Dy^3+^	^6^H_9/2_ + ^6^F_11/2_ → ^6^H_15/2_	1328	68	97.1	
	^3^H_4_ → ^3^F_4_	1470	145	99.7	
	^3^F_4_ → ^3^H_6_^6^H_11/2_ → ^6^H_15/2_	1794	847	99.9	
	^3^H_4_ → ^3^H_5_	2316	163	98.87	
	^6^H_13/2_ → ^6^H_15/2_	2887	2829	99.6	
Er^3+^-Dy^3+^	^6^H_9/2_ + ^6^F_11/2_ → ^6^H_15/2_	1328	39	90.1	
	^4^I_13/2_ → ^4^I_15/2_	1540	3218	92.3	
	^6^H_11/2_ → ^6^H_15/2_	1752	442	99.5	
	^6^H_13/2_ → ^6^H_15/2_	2887	1973	97.4	
Tm^3+^-Er^3+^-Dy^3+^	^6^H_9/2_ + ^6^F_11/2_ → ^6^H_15/2_	1328	79	99.4	
	^3^H_4_ → ^3^F_4_	1470	140	99.4	
	^4^I_13/2_ → ^4^I_15/2_	1540	2551	97.7	27.5
	^3^F_4_ → ^3^H_6_^6^H_11/2_ → ^6^H_15/2_	1794	744	99.9	
	^3^H_4_ → ^3^H_5_	2316	137	99.6	
	^6^H_13/2_ → ^6^H_15/2_	2887	1654	99.6	
