# Supplementary material for: GeS2–In2S3–CsI Chalcogenide Glasses Doped with Rare Earth Ions for Near- and Mid-IR Luminescence
Source: Sci Rep. 2016 Nov 21;6:37577. doi: 10.1038/srep37577 (PMC5116617; doi:10.1038/srep37577)
Supplement: Supplementary Information [file srep37577-s1.pdf]

GeS_2–In_2S_3–CsI Chalcogenide Glasses Doped with Rare Earth Ions for Near- and Mid-IR Luminescence

Legang Li,[1,2] Junyi Bian,[1,2] Qing Jiao,[1,2] Zijun Liu,[1,2] Shixun Dai,[1,2] Changgui Lin[1,2,*]

[1]Laboratory of Infrared Materials and Devices, The Research Institute of Advanced Technologies, Ningbo University, Ningbo 315211, China

[2]Key Laboratory of Photoelectric Detection Materials and Devices of Zhejiang Province, Ningbo University, Ningbo 315211, China

***Corresponding author:** linchanggui@gmail.com (C. Lin)

Supplementary Table S1. Energy transfer efficiency of all samples

Sample	Transition	Wavelength (nm)	Transfer efficiency η (%)
Tm^{3+}-Er^{3+}	Tm^{3+}:$^3H_4$,Er^{3+}:$^4I_{15/2}$→Tm^{3+}:$^3H_6$,Er^{3+}:$^4I_{9/2}$	1470	9.3
	Er^{3+}:$^4I_{13/2}$,Tm^{3+}:$^3H_6$→Er^{3+}:$^4I_{15/2}$,Tm^{3+}:$^3H_5$	1540	28.2
	Tm^{3+}:$^3H_4$,Er^{3+}:$^4I_{15/2}$→Tm^{3+}:$^3H_6$,Er^{3+}:$^4I_{9/2}$	2316	1.2
Tm^{3+}-Dy^{3+}	Tm^{3+}:$^3H_4$,Dy^{3+}:$^6H_{15/2}$→Tm^{3+}:$^3H_6$,Dy^{3+}:$^6F_{5/2}$	1470	15.6
	Tm^{3+}:$^3F_4$,Dy^{3+}:$^6H_{15/2}$→Tm^{3+}:$^3H_6$,Dy^{3+}:$^6H_{11/2}$	1804	28.7
	Tm^{3+}:$^3H_4$,Dy^{3+}:$^6H_{15/2}$→Tm^{3+}:$^3H_6$,Dy^{3+}:$^6F_{5/2}$	2316	7.9
Tm^{3+}-Er^{3+}-Dy^{3+}	Er^{3+}:$^4I_{13/2}$,Tm^{3+}:$^3H_6$+ Dy^{3+}:$^6H_{15/2}$ →Er^{3+}:$^4I_{15/2}$,Tm^{3+}:$^3F_4$+ Dy^{3+}:$^6H_{11/2}$	1540	27.5

Supplementary Table S2. The reduction and improvement of the emissions

Sample	Transition	Wavelength (nm)	Transfer efficiency β (%)	Change
Tm^{3+}-Er^{3+}	$^3H_4 \rightarrow ^3F_4$	1470	32.8	reduction
	$^4I_{13/2} \rightarrow ^4I_{15/2}$	1540	79	improvement
	$^3F_4 \rightarrow ^3H_6$	1804	19.5	improvement
	$^3H_4 \rightarrow ^3F_4$	2316	11	reduction
Tm^{3+}-Dy^{3+}	$^3H_4 \rightarrow ^6F_{5/2}$	1328	17	improvement
	$^3F_4 \rightarrow ^3H_6$<br>$^6H_{11/2} \rightarrow ^6H_{15/2}$	1794	398	improvement
	$^6H_{13/2} \rightarrow ^6H_{15/2}$	2887	18	improvement
Er^{3+}-Dy^{3+}	$^6H_{9/2}+^6F_{11/2}$<br>$\rightarrow ^6H_{15/2}$	1328	16.7	reduction
	$^4I_{13/2} \rightarrow ^4I_{15/2}$	1540	35.2	reduction
	$^6H_{11/2} \rightarrow ^6H_{15/2}$	1752	6.8	reduction
	$^6H_{13/2} \rightarrow ^6H_{15/2}$	2887	22.4	reduction
Tm^{3+}-Er^{3+}-Dy^{3+}	$^4I_{13/2} \rightarrow ^4I_{15/2}$	1328	7.4	improvement
	$^3H_4 \rightarrow ^3F_4$	1470	18.2	improvement
	$^4I_{13/2} \rightarrow ^4I_{15/2}$	1540	28.8	reduction
	$^3F_4 \rightarrow ^3H_6$<br>$^6H_{11/2} \rightarrow ^6H_{15/2}$	1794	26.7	improvement
	$^3H_4 \rightarrow ^3F_4$	2316	11.6	improvement
	$^6H_{13/2} \rightarrow ^6H_{15/2}$	2887	4.7	improvement

The co-doped samples are compared to the single-doped samples and the triple-doped sample is compared to Tm^{3+}-Dy^{3+} co-doped and Er^{3+}-doped sample.
